# Impact of early life stress on the immune response and bone regeneration after fracture

**DOI:** 10.1016/j.bonr.2026.101932

**Published:** 2026-06-23

**Authors:** Dorothea Gebauer, Tamara Schimmele, Dominik Langgartner, Jana Bleher, Anita Ignatius, Stefan O. Reber, Melanie Haffner-Luntzer

**Affiliations:** a Institute of Orthopaedic Research and Biomechanics, Ulm University Medical Center, 89081, Ulm, Germany; b Laboratory for Molecular Psychosomatics, Department of Psychosomatic Medicine and Psychotherapy, Ulm University Medical Center, 89081, Ulm, Germany; c German Center for Mental Health (DZPG), partner site Mannheim//-Heidelberg//-Ulm, Germany

**Keywords:** Early life stress (ELS), Psychosocial stress, Maternal separation (MS), Femur osteotomy, Fracture healing

## Abstract

Early-life stress (ELS) constitutes an enormous socio-economic burden and is an acknowledged risk factor for the development of several affective and somatic conditions, including bone disorders. However, its effects on bone fracture healing have not been studied yet. Here, we implemented a novel two-hit model in male and female C57BL/6N mice, where we used maternal separation (MS) to induce ELS and combined it with a standardized right femur osteotomy stabilized by an external fixator. Behavior was analyzed using the Open Field/Novel Object test, Social Preference/Avoidance test and Saccharin Preference test. Fracture healing was evaluated by biomechanical testing, μCT, histology, and cytokine assays. Maternal separation let to an altered inflammatory response 3 h after fracture, indicated by a significant reduction in several pro- and anti-inflammatory cytokines. This effect was more pronounced in male mice. However, later fracture phases (10 days and 21 days after osteotomy) showed no significant differences between stressed and control mice. Our results indicate that ELS might alter inflammatory responses towards bone fracture in a sex-specific manner without disturbing fracture healing.

## 1 Introduction

Early life stress (ELS) affects about 39% of the global population (Elwenspoek et al., 2017; Kessler et al., 2010) and is recognized as a critical risk factor for a variety of mental and somatic disorders, including posttraumatic stress disorder (PTSD), major depression, and obesity (Kuzminskaite et al., 2020; Felitti et al., 2019; Hughes et al., 2017; Maercker et al., 2019), with evidence suggesting women to be in general more susceptible than men (Danese and Tan, 2014). ELS is known to promote chronic low-grade inflammation (Elwenspoek et al., 2017; Boeck et al., 2016; Miller et al., 2009), which is characterized, among others, by elevated plasma levels of proinflammatory cytokines, C-reactive protein and fibrinogen, alongside with increased white blood cell counts (Danese et al., 2007; Surtees et al., 2003; Baumeister et al., 2016).

Our group has demonstrated in a series of recent studies that an exaggerated inflammatory response triggered by chronic psychosocial stress in adulthood markedly impairs long-bone growth (Foertsch et al., 2017) and bone regeneration following fracture (Haffner-Luntzer et al., 2019) in male mice. This process is mediated by stress-activated myeloid immune cells producing and secreting catecholamines and inflammatory cytokines locally in the bone marrow and fracture hematoma (Tschaffon-Muller et al., 2023). Expanding on this, own studies further showed that ELS has profound and sex-specific effects on bone health. Specifically, maternal separation (MS)-induced ELS increased osteoclast activity and proinflammatory cytokine expression predominantly in female mice, associated with microbiome alterations, sustained immune activation and a catabolic bone turnover leading to an osteopenic bone phenotype (Schimmele et al., 2025). Complementary findings confirmed that bones from female mice are more susceptible to the negative effects of ELS, while bones from male mice exhibit stronger responses to chronic adult psychosocial stress (CAS) (Gebauer et al., 2025).

Fracture healing is a multifaceted biological process that unfolds through several overlapping phases, beginning with an inflammatory phase immediately following injury (Claes et al., 2012; Kolar et al., 2010). This process depends on the tightly regulated proliferation, migration and differentiation of various cell types, including inflammatory cells, angioblasts, fibroblasts, chondrocytes, and osteoblasts (Wang et al., 2024). A well-regulated inflammatory response is essential for effective bone repair (Haffner-Luntzer et al., 2017; Prystaz et al., 2018; Kovtun et al., 2016). During this phase, a hematoma forms at the injury site, recruiting immune cells, which in turn release cytokines and growth factors essential for initiating tissue repair (Claes et al., 2012; Kolar et al., 2010). The subsequent reparative phase involves soft callus formation, which gradually mineralizes into bone callus, followed by the remodeling phase that reconstructs the bone's architecture (Claes et al., 2012; Matthews et al., 2014; Shi et al., 2017).

Given that ELS promotes chronic low-grade inflammation and negatively impacts bone metabolism in female mice, we hypothesized in the present study that ELS will sex-specifically disturb the tightly regulated immune response required for adequate fracture healing and, thus, compromise bone regeneration and repair after injury. To test this, male and female offspring of C57BL/6N mothers have been exposed to the MS (postnatal days (PNDs) 1–14 for 3 h per day) paradigm and subsequently to experimental femur osteotomy at the age of 12 weeks. Animals were euthanized at three different time points (3 h, 10 d and 21 d post-osteotomy) to investigate distinct phases of fracture healing.

## 2 Material and methods

### 2.1 Animals

Breeding male and female C57BL/6N mice were obtained from Charles River (Sulzfeld, Germany) and used to generate in-house bred C57BL/6N offspring. All mice were housed in standard polycarbonate cages (16 cm width x 22 cm length x 14 cm height) under specific pathogen-free (SPF) laboratory conditions. The housing environment was maintained at 22 °C with 60% humidity and a 12-h light/dark cycle (lights on at 06:00 a.m. in winter; 07:00 a.m. in summer). Animals had free access to tap water and a standard mouse diet. All experimental procedures were conducted in compliance with the ARRIVE 2.0 guidelines (Percie du Sert et al., 2020) and approved by the Federal Committee (Regierungspräsidium Tübingen, Germany). Every effort was made to minimize animal numbers and distress.

### 2.2 Experimental procedure

**Fig. 1:**
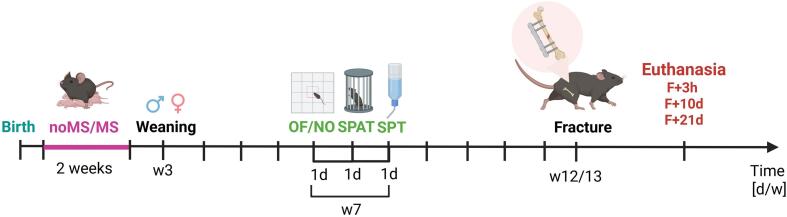
Experimental timeline. Maternal separation (MS) was carried out from postnatal day (PND) 1 to 14, with unseparated offspring (noMS) serving as controls (i.e., remained undisturbed, apart from daily routine handling between PNDs 1 and 14). The offspring were weaned from their dams on PND 21 and experimental male and female mice were group-housed according to treatment and sex, with 3–4 animals per cage. With an age of seven weeks (w), all experimental mice were exposed to the open-field/novel object (OF/NO) test, the social preference/avoidance test (SPAT) and the saccharin preference test (SPT). At 12–13 w of age, mice underwent unilateral femur osteotomy and were euthanized either 3 h (h), 10 days (d) or 21 d post-fracture. The illustration was created with Biorender.com.

The experimental procedure is illustrated in Fig. 1. Pregnant females were monitored daily. The day of birth was designated as postnatal day (PND) 0 if delivery occurred before 02:00 p.m.; if birth took place after 02:00 p.m., the following day was assigned as PND 0. On PND 1, dams and their litters were randomly assigned to either the “no maternal separation” (noMS) group or the “maternal separation” (MS) group, before being exposed to their respective conditions daily until PND 14. Afterwards, the litters remained undisturbed with their dams until weaning at PND 21. After weaning, experimental male and female mice were housed in same-sex groups according to their treatment, with 3–4 animals per cage. At 7 weeks of age, all experimental mice underwent a series of behavioral tests on consecutive days, starting with the open-field/novel object (OF/NO) test to assess general anxiety-like behavior, followed by the social preference/avoidance test (SPAT) to assess general and social anxiety-like behavior as well as the saccharin preference test (SPT, 24 h) to assess anhedonia-like behavior. At 12–13 weeks of age, mice underwent unilateral femur osteotomy. Therefore, mice were housed individually the day before surgery and the days following surgery. Animals were euthanized either 3 h, 10 d or 21 d post-fracture. Except for the 3 h time point, all animals were euthanized in the mornings by rapid decapitation following brief CO_2_ inhalation.

### 2.3 Maternal separation (MS)

The MS procedure was conducted in line with previous studies (Schimmele et al., 2025; Mazzari et al., 2023; Veenema et al., 2008). In brief, from PND 1–14, pups were separated from their dams for 3 h per day (wintertime: 08:00–11:00 a.m.; summertime: 09:00 a.m. – 12:00 p.m.). At the start of each separation, dams were removed from the maternity cage and placed individually into holding cages, while the entire litter was transferred into a small box on a heating pad (30–33 °C) in an adjacent room. After the 3 h-separation period, pups were returned to their home cage and reunited with the dam. Control offspring (noMS) remained with their dam and were left undisturbed, apart from routine daily handling during PNDs 1–14. Body weight of dams and litter weight were recorded weekly, and bedding was changed at PND 14.

### 2.4 Open field/novel object (OF/NO) test

To assess effects on general anxiety-related behavior, the OF/NO test was conducted at the age of seven weeks between 07:00 a.m. and 10:00 a.m. as previously described (Schimmele et al., 2025; Mazzari et al., 2023; Amoroso et al., 2020). In detail, the test arena (45 cm length × 27 cm width × 27 cm height; 350 lx) was subdivided into an inner (9 × 27 cm), and an outer zone. The mouse was placed into the inner zone and allowed to explore the arena for 5 mins. After 5 min of OF exploration, a round-shaped NO (diameter: 3.5 cm; height: 1.5 cm) was placed into the middle of the inner zone. The mouse was then allowed to explore the arena containing the NO for 5 min. During OF exploration, the total distance moved as well as time spent in inner zone were analyzed. Furthermore, during NO exploration, total distance moved and time in contact zone were analyzed. The test arena was cleaned thoroughly with water after each trial. All parameters were analyzed using EthoVision XT (v11.5.1022; Noldus Information Technology, Wageningen, The Netherlands).

### 2.5 Social preference/avoidance test (SPAT)

To investigate effects on general and social anxiety-related behavior, the SPAT was conducted the day after the OF/NO test between 07.00 a.m. and 10.00 a.m. as described previously (Mazzari et al., 2023; Schiele et al., 2026). Briefly, the experimental mouse was placed into the arena (45 cm length × 27 cm width × 27 cm height; 20 lx) for 30 s habituation to the unfamiliar environment before a small empty wire mesh cage (8.5 cm length x 7.5 cm width x 6.5 cm height) was introduced for 150 s. Afterwards, the empty cage was exchanged with an identical cage containing an unfamiliar C57BL/6N mice (male experimental mice were exposed to an unfamiliar male conspecific, female experimental mice were exposed to an unfamiliar female conspecific) for another 150 s. The total distance moved and time spent in direct contact zone were analyzed using EthoVision XT (v11.5.1022; Noldus Information Technology, Wageningen, The Netherlands). The box was cleaned thoroughly with water following every test.

### 2.6 Saccharin preference test (SPT)

For the assessment of anhedonia-like behavior, the SPT was performed one day after the SPAT as previously described (Schiele et al., 2026; Slattery et al., 2012). Briefly, experimental mice were habituated to the option of two drinking bottles filled with tap water, starting immediately after the OF/NO test. During the SPT, all experimental mice were single-housed and simultaneously exposed to one bottle containing 0.05% saccharine solution (Sigma-Aldrich, Chemie GmbH, Steinheim, Germany) and one bottle containing tap water for 24 h. To exclude side preferences, bottles containing the saccharin solution and tap water were switched repeatedly. The weights of both bottles were determined at the beginning and end of the SPT. The saccharin preference in percent was calculated based on the following calculation:

$$\text{Saccharin intake}\;\left[g\right]/\left(\text{saccharin intake}\;\left[g\right]+\text{water intake}\;\left[g\right]\right)\times 100$$

Of note, 3 MS males and 1 noMS female had to be excluded from the analysis due to leakage of the test bottles.

### 2.7 Femur osteotomy

At an age of 12 weeks, a standardized right femur osteotomy stabilized with an external fixator was performed as described previously (Rontgen et al., 2010). For analgesia, mice received tramadol hydrochloride (0,1 mg/ml; Gruenenthal GmbH, Aachen, Germany) in their drinking water 1 day pre- till 3 days post-surgery, and as a subcutaneous injection (25 mg/kg body weight; Libra-Pharm GmbH, Aachen, Germany) immediately before surgery. Additionally, mice were subcutaneously injected with Clindamycin (45 mg/kg body weight; MIP Pharma GmbH, Blieskastel, Germany) and 500 μl sodium chloride (Fresenius Kabi, Bad Homburg, Germany). Anesthesia was performed using 2% isoflurane (Baxter International, Deerfield, IL, USA). Mice were allowed to move freely after surgery. To analyze different phases of fracture healing, mice were sacrificed 3 h, 10 days and 21 days after osteotomy.

### 2.8 Twenty-four hour home cage locomotion

The 24 h home cage locomotion was assessed on d1 after femur osteotomy. All mice were videotaped (wintertime: 06:00 p.m. - 06:00 p.m.; summertime: 07:00 p.m. – 07:00 p.m.) in their home cage from above and the distance moved was analyzed using EthoVision XT (version 11).

### 2.9 Trunk blood sampling

Trunk blood was collected in EDTA-coated tubes (Sarstedt, Nuembrecht, Germany) following short CO_2_ inhalation and decapitation as previously published (Schimmele et al., 2025; Amoroso et al., 2019). Tubes were centrifuged at 4 °C (5000 *g*, 10 min) before plasma of each individual mouse was stored at −20 °C until further use.

### 2.10 Multiplex cytokine analysis

To determine cytokine levels in the early inflammatory phase of fracture healing, a ProcartaPlex™ 36plex (Bender MedSystems GmbH, Vienna, Austria) assay was used to determine cytokine concentration in the fracture hematoma and plasma samples of animals sacrificed 3 h after fracture. Concentration values below the detection limit were set to the lowest measurable standard.

### 2.11 Biomechanical testing

Femurs were dissected, cleaned, and stored in a sodium chloride solution. To evaluate the flexural rigidity of the healed fracture callus 21 days post-surgery, a non-destructive three-point bending test was performed as described previously (Rontgen et al., 2010). Bending stiffness was determined for the right fractured femur itself (absolute value), and for the right femur in relation to the contralateral unfractured femur (relative value). After measurement, femurs were transferred into 4% formalin for further μCT analysis.

### 2.12 μCT analysis

For structural assessment 21 days post-surgery, right femurs were scanned using the Skyscan 1172 scanning device (Skyscan, Kontich, Belgium), operating at a voxel resolution of 8 μm and at 200 μA and 50 kV. Two phantoms with a defined hydroxyapatite (HA) density (250 and 750 mg/cm^3^) were included within each scan. Using Skyscan software (NRecon, DataViewer, CTAn, and CTVox), analysis was conducted according to ASBMR guidelines (Bouxsein et al., 2010). As volume of interest (VOI), the fracture callus within the inner two pin holes was analyzed. A threshold of 642 mg HA/cm^3^ was used to distinguish between mineralized and non-mineralized tissue, as described previously (Morgan et al., 2009).

### 2.13 Histological analysis

Right femurs were subjected to decalcified histology and embedded in paraffin, as described previously (Gebauer et al., 2025). Longitudinal sections of 4 μm were stained using tartrate-resistant acid phosphatase (TRAP) for osteoclast or Safranin O/Fast Green for osteoblast parameters. Slides were examined at a magnification of 20 using light microscopy (Axiophot, Zeiss, Oberkochen, Germany), and analysis was conducted using the OsteoMeasure software (Osteometrics, Decatur, USA) as described previously (Haffner-Luntzer et al., 2016).

Using an image analysis software (Leica MMAF 1.4.0 Imaging System, Leica, Herbrugg, Switzerland), the relative amounts of bone, cartilage, and connective tissue of the fracture callus located between the two inner pinholes were determined according to ASBMR guidelines (Dempster et al., 2013).

### 2.14 Statistical analysis

For statistical analysis and graphical illustrations GraphPad Prism (version 11.0.0, GraphPad Software, LCC) was used as described previously (Tschaffon-Muller et al., 2023; Schimmele et al., 2025). In detail, Kolmogorov-Smirnov test with Lilliefors' correction was employed to test for normal distribution and normally distributed data sets were tested for one extreme outlier per group by using Grubbs test (Grubbs, 1969): The outliers were excluded from further analysis. Normally distributed data sets were further analyzed by two-tailed Student's *t*-tests (one factor, two independent datasets) with Welch's correction when appropriate. Not normally distributed data sets were analyzed by non-parametric statistics, i.e. Mann-Whitney *U* test (one factor, two independent datasets) or Wilcoxon matched-pairs signed rank test (one factor, two dependent samples). Data are presented as box plots (median (thick line); mean (plus sign); 25th and 75th percentiles; minimum and maximum values, outliers) including individual values. The level of significance was set at *P* ≤ 0.05.

## 3 Results

### 3.1 Effects of MS on general and social anxiety-related behavior

**Fig. 2:**
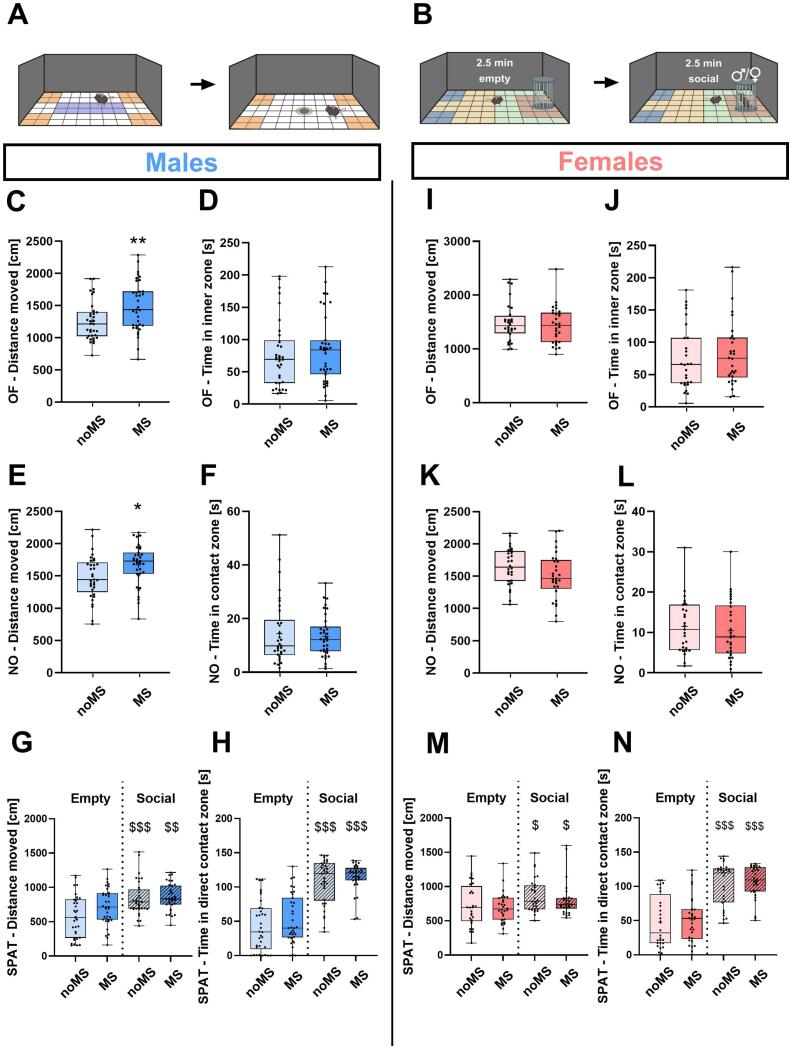
Effects of maternal separation (MS) on general and social anxiety-related behavior. Experimental setup for the (A) open field/novel object (OF/NO) test and (B) social preference/avoidance test (SPAT). (C–H) Males. (I–N) Females. (C/I) Total distance moved during OF exploration. (D/J) Time spent in inner zone during OF exploration. (E/K) Total distance moved during NO exploration. (F/L) Time spent in contact zone during NO exploration. (G/M) Total distance moved during SPAT exposure. (H/N) Time in direct contact zone during SPAT exposure. Data are presented as box-plots including individual values, with solid line representing the median and plus sign representing the mean for each data-set. Lower boxes indicate 25th, upper boxes 75th percentile; minimum (lower error bar), maximum (upper error bar) as well as possible outliers (closed circles beyond the percentiles) are also shown. *P ≤ 0.05, **P ≤ 0.01 versus noMS condition; ^$^*P* ≤ 0.05, ^$$^*P* ≤ 0.01, ^$$$^*P* ≤ 0.001 versus respective empty cage condition. Males: noMS, *N* = 33–34, MS, *N* = 35; Females: noMS, *N* = 27–28, MS, *N* = 30.

In males, total distance moved was significantly increased in MS vs. noMS mice during OF exploration (Fig. 2C; Student's *t*-test: *P* = 0.008), during NO exploration (Fig. 2E; Student's t-test: *P* = 0.019), and by trend (Fig. 2G; MWU: *P* = 0.060) during empty cage exploration in the SPAT (Fig. 2G; MWU: *P* = 0.060). Time spent in the inner zone during OF exploration and in the contact zone during NO exploration were comparable between the groups. Total distance moved (Fig. 2G; MS, Wilcoxon test: *P* = 0.001; noMS, Wilcoxon test: *P* < 0.001) as well as the time spent in direct contact with the social stimulus (Fig. 2H; MS, Wilcoxon test: *P* < 0.001; noMS, Wilcoxon test: *P* = 0.001) were further increased during social vs. empty cage exploration in both MS and noMS mice.

In females, total distance moved (OF + NO condition) and time spent in the inner zone during OF exploration as well as time in contact zone during NO exploration were comparable between the groups (Fig. 2I–L). During SPAT exposure, total distance moved (Fig. 2M; MS, Wilcoxon test: *P* = 0.025; noMS, Wilcoxon test: *P* = 0.036) and time in direct contact zone (Fig. 2N; MS, Wilcoxon test: *P* < 0.001; noMS, Wilcoxon test: *P* < 0.001) were increased during social vs. empty cage exploration.

### 3.2 Effects of MS on anhedonia-like behavior

In males and females, statistical analysis revealed that total fluid intake (Mean ± SEM: Males: noMS 13.18 ± 0.36, MS 13.33 ± 0.46; Females: noMS 15.91 ± 0.89, MS 14.86 ± 0.64) and saccharin preference (Males: noMS 0.64 ± 0.02, MS 0.64 ± 0.01; Females: noMS 0.67 ± 0.01, MS 0.69 ± 0.01) were comparable between groups.

### 3.3 Impact of MS on fracture healing during the inflammation phase (3 h post-fracture)

**Fig. 3:**
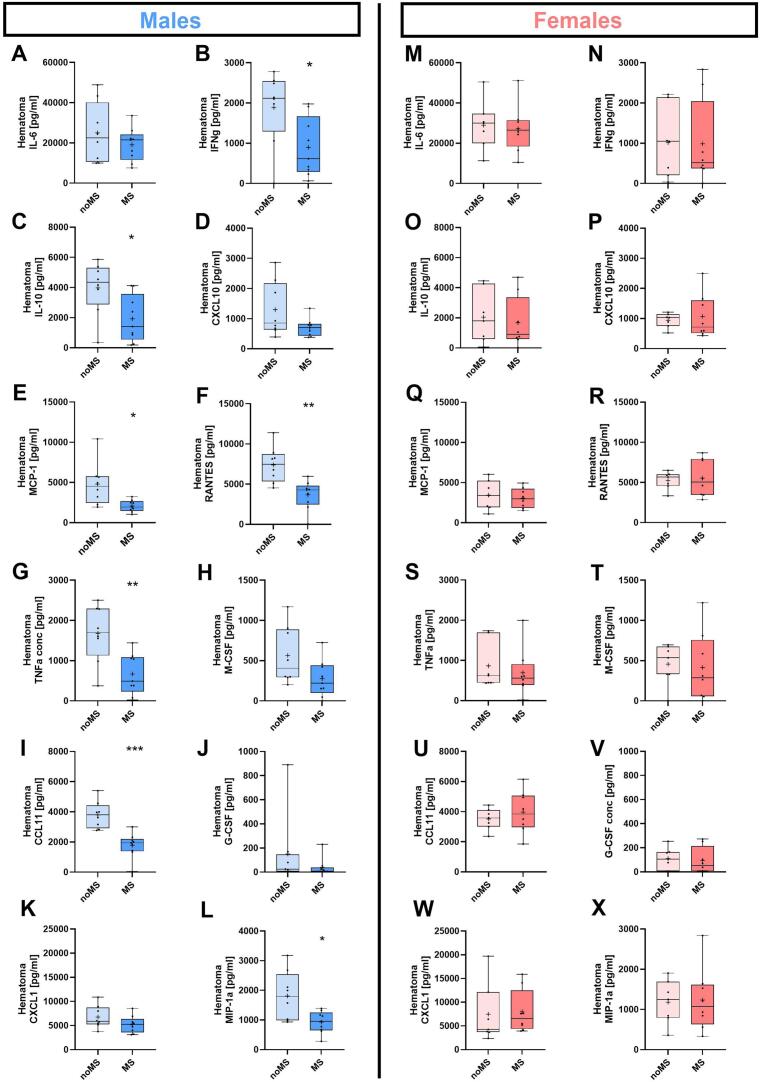
Effects of maternal separation (MS) on fracture hematoma cytokine concentrations during the inflammation phase (3 h post-fracture). (A–L) Males. (M–X) Females. (A/M) Interleukin 6 (IL-6). (B/N) Interferon gamma (IFNg). (C/O) IL-10. (D/P) C-X-C motif chemokine ligand 10 (CXCL10). (E/Q) Monocyte chemoattractant protein-1 (MCP-1). (F/R) Regulated upon activation, normal T cell expressed and secreted (RANTES). (G/S) Tumor necrosis factor alpha (TNFα). (H/T) Macrophage colony stimulating factor (M-CSF). (I/U) C-C motif chemokine 11 (CCL11). (J/V) Granulocyte-colony stimulating factor (G-CSF). (K/W) C-X-C motif chemokine ligand 1 (CXCL1). (L/X) Macrophage inflammatory protein 1a (MIP-1a). Data are presented as box-plots including individual values, with solid line representing the median and plus sign representing the mean for each data-set. Lower boxes indicate 25th, upper boxes 75th percentile; minimum (lower error bar), maximum (upper error bar) as well as possible outliers (closed circles beyond the percentiles) are also shown. *P ≤ 0.05, **P ≤ 0.01 versus noMS condition. Males: noMS: *N* = 7–8, MS: *N* = 8–9; Females: noMS: *N* = 7–8, MS: *N* = 7–8.

In males, **hematoma levels** (Fig. 3) of IFN-γ (Fig. 3B; MWU: *P* = 0.036), IL-10 (Fig. 3C; Student's *t*-test: *P* = 0.023), MCP-1 (Fig. 3E; Student's t-test: *P* = 0.011), RANTES (Fig. 3F; Student's t-test: *P* = 0.002), TNF-α (Fig. 3G; Student's t-test: *P* = 0.004), CCL11 (Fig. 3I; Student's *t*-test: *P* < 0.001) and MIP-1a (Fig. 3L; Student's *t*-test: *P* = 0.036) were significantly reduced in MS vs. noMS mice, while M-CSF was reduced by trend (Fig. 3H; Student's t-test: *P* = 0.060). Statistical analysis further revealed that **hematoma levels** of IL-6 (Fig. 3A), G-CSF (Fig. 3J), and CXCL1 (Fig. 3K) remained unaffected by MS. **Plasma levels** (Table 1) of CXCL10 (Student's t-test: *P* = 0.003) and RANTES (Student's t-test: *P* = 0.028) were significantly reduced in MS vs. noMS mice, while CXCL1 was only by trend reduced (Student's t-test: *P* = 0.060). **Plasma levels** (Table 1) of IL-6, IL-10, MIP-1a, TNF-α, M-CSF, CCL11 and G-CSF were comparable between groups, while IFN-γ and MCP-1 levels were undetectable.

**Table 1:** Plasma cytokine concentrations.

	noMS [pg/ml]	MS [pg/ml]
*Plasma cytokines, males*		
IL-6	560.3 ± 149.6	425.4 ± 140.3
IFN-γ	n.d.	n.d.
IL-10	30.4 ± 7.4	23.4 ± 2.1
CXCL10	86.6 ± 10.8	48.6 ± 10.6**
MCP-1	n.d.	n.d.
MIP-1a	8.7 ± 2.4	8.2 ± 2.1
RANTES	251.9 ± 33.1	141.9 ± 30.8*
TNF-α	15.3 ± 1.9	17.4 ± 1.6
M-CSF	150.0 ± 86.3	75.7 ± 26.0
CCL11	924.4 ± 221.4	595.9 ± 180.7
G-CSF	32.1 ± 9.9	24.9 ± 7.8
CXCL1	2075.0 ± 440.4	1108.5 ± 219.2

*Plasma cytokines, females*		
IL-6	408.9 ± 90.0	230.0 ± 88.4
IFN-γ	n.d.	n.d.
IL-10	n.d.	n.d.
CXCL10	13.5 ± 13.9	47.9 ± 22.2
MCP-1	n.d.	n.d.
MIP-1a	n.d.	n.d.
RANTES	267.7 ± 32.0	163.4 ± 50.5
TNF-α	19.8 ± 0.8	17.3 ± 1.7
M-CSF	n.d.	n.d.
CCL11	821.5 ± 99.7	306.3 ± 130.1*
G-CSF	24.4 ± 6.4	7.4 ± 0.6*
CXCL1	1470.3 ± 252.5	362.5 ± 151.3**

Abbreviations: CCL11, C-C motif chemokine 11; CXCL1, C-X-C motif chemokine ligand 1; CXCL10, C-X-C motif chemokine ligand 10; G-CSF, granulocyte-colony stimulating factor; IL, interleukin; IFN, interferon; MCP-1, monocyte chemoattractant protein-1; M-CSF, macrophage colony-stimulating factor; MIP-1a, macrophage inflammatory protein 1a; MS, maternal separation; n.d., not determined; noMS, no maternal separation; RANTES, regulated on activation, normal T cell expressed and secreted; TNF-α, tumor necrosis factor α. *P ≤ 0.05, **P ≤ 0.01 versus noMS condition. Males: noMS: *N* = 7–8, MS: *N* = 8–9; females: noMS: *N* = 7–8, MS: *N* = 7–8.

In females, **hematoma levels** of IL-6, IFN-γ, IL-10, CXCL10, MCP-1, RANTES, TNF-α, M-CSF, CCL11, G-CSF, CXCL1 and MIP-1a (Fig. 3M–X) remained unaffected by maternal separation. **Plasma levels** (Table 1) of CCL11 (Student's t-test: *P* = 0.020), G-CSF (Student's t-test: *P* = 0.011) and CXCL1 (Student's t-test: *P* = 0.003) were significantly reduced in MS vs. noMS mice. **Plasma levels** (Table 1) of IL-6, CXCL10, RANTES and TNF-α were comparable between groups, while IFN-γ, IL10, MCP-1, MIP-1a and M-CSF levels were undetectable.

### 3.4 Impact of MS on fracture healing during the intermediate repair phase (10 d post-fracture)

**Fig. 4:**
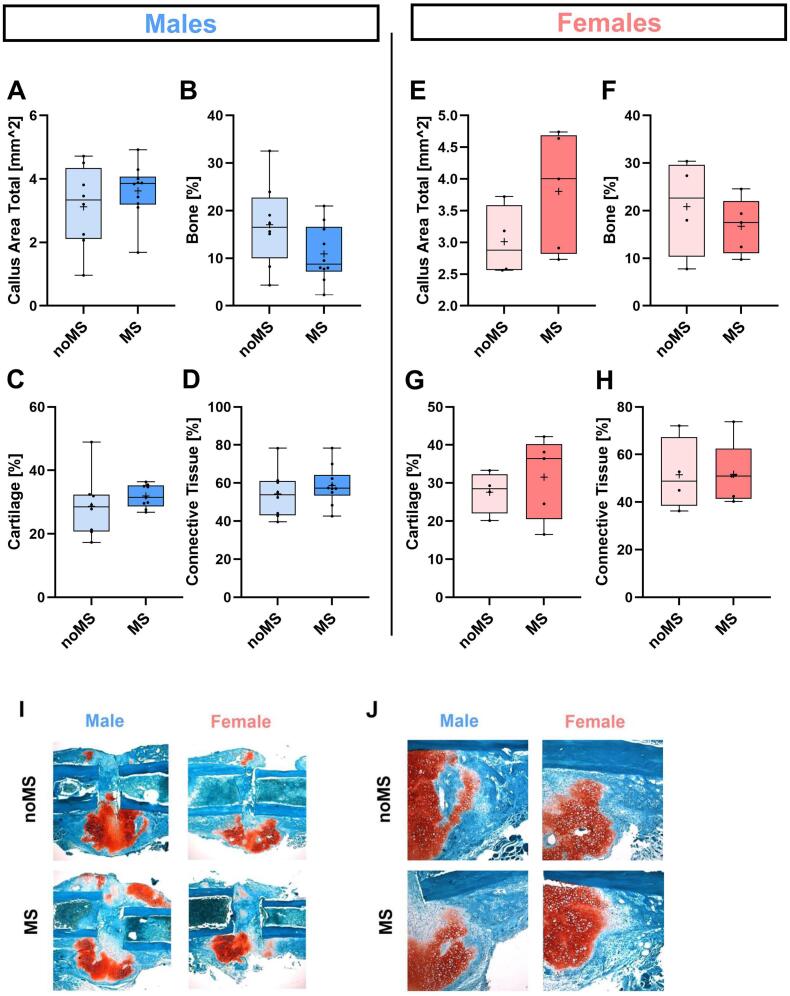
Impact of maternal separation (MS) on fracture healing during the repair phase (day 10 post-fracture). (A–D) Males. (E–H) Females. (A/E) Total callus area. (B/F) % of bone in the callus. (C/G) % of cartilage in the callus. (D/H) % of connective tissue in the callus. (I/J) Representative histological images showing cartilage (blue) and bone (red) regions in the callus stained with Safranin O. Data are presented as box-plots including individual values, with solid line representing the median and plus sign representing the mean for each data-set. Lower boxes indicate 25th, upper boxes 75th percentile; minimum (lower error bar), maximum (upper error bar) as well as possible outliers (closed circles beyond the percentiles) are also shown. Males: noMS *N* = 8, MS *N* = 9–10; Females: noMS *N* = 4, MS *N* = 5.

**Supplemental Fig. 1:**
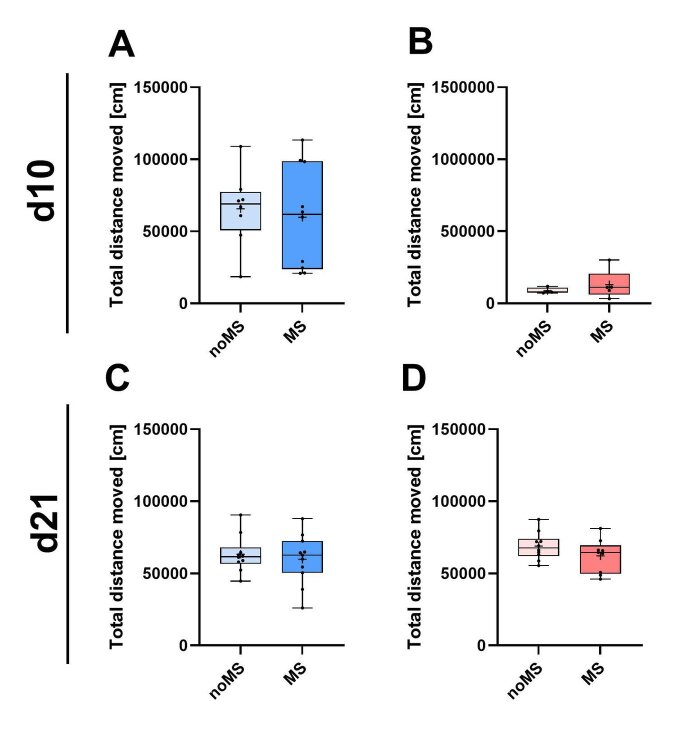
24-h home cage locomotion following femur osteotomy (A/C) Males. (B/D) Females. (A/B) Distance moved during 24-h home cage locomotion tracking in noMS and MS mice on the day of the femur osteotomy of d10 sacrificed mice. (C/D) Distance moved during 24-h home cage locomotion tracking in maternal separation (MS) and noMS mice on the day of the femur osteotomy of d21 sacrificed mice. Data are presented as box-plots including individual values, with solid line representing the median and plus sign representing the mean for each data-set. Lower boxes indicate 25th, upper boxes 75th percentile; minimum (lower error bar), maximum (upper error bar) as well as possible outliers (closed circles beyond the percentiles) are also shown. Males d10: noMS *N* = 8, MS *N* = 10; Males d21: noMS *N* = 10, MS *N* = 11; Females d10: noMS *N* = 4, MS *N* = 5; Females d21 noMS *N* = 10, MS *N* = 9.

In males (Fig. 4A–D) and females (Fig. 4E–H), statistical analysis revealed that the total callus area (Fig. 4A/E), the percentage of the bone (Fig. 4B/F), cartilage (Fig. 4C/G) and connective tissue (Fig. 4D/H) of the callus were comparable between groups. To ensure that potential effects on fracture healing were not mediated by differences in general locomotor activity, 24-h home cage activity was assessed directly after surgery. Importantly, no differences between noMS and MS mice were measured for either sex (Fig. S1A/B).

### 3.5 Impact of MS on fracture healing during the late repair phase (21 d post-fracture)

**Fig. 5:**
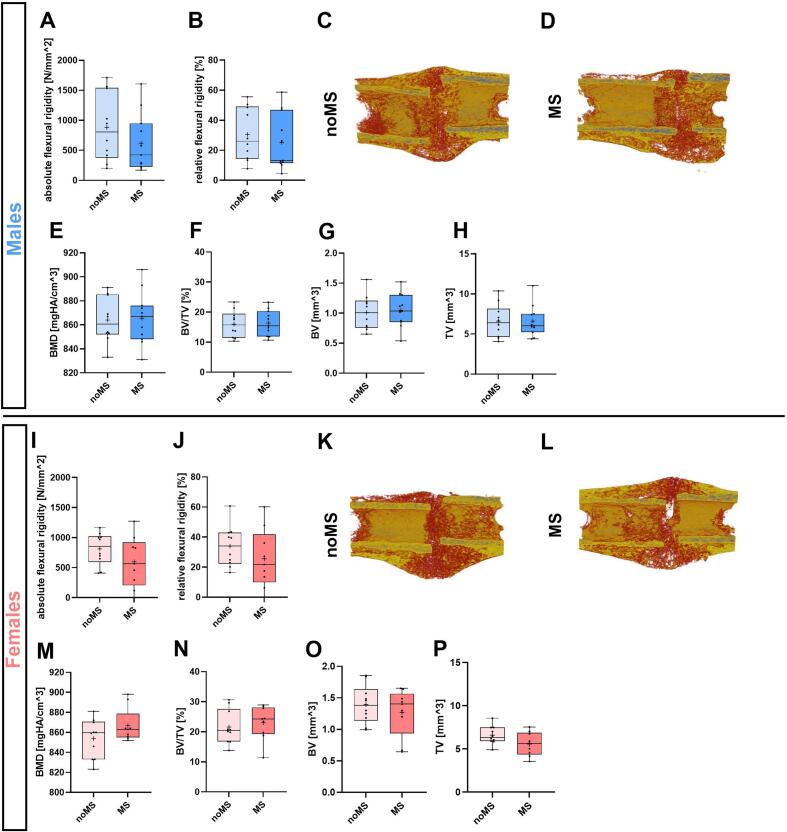
Impact of maternal separation (MS) on bone stability and microarchitecture during the remodeling phase of fracture healing (day 21 post-fracture). (A–H) Males. (I–P) Females. (A/I) Absolute flexural rigidity. (B/J) Relative flexural rigidity. (C–D/K–L) Representation of 3D images of the fracture callus in the analyzed region of interest of noMS (C/K) and MS (D/L) animals. (E/M) Bone mineral density (BMD). (F/N) Bone volume/tissue volume (BV/TV). (G/O) Bone volume (BV). (H/P) Tissue volume (TV). Data are presented as box-plots including individual values, with solid line representing the median and plus sign representing the mean for each data-set. Lower boxes indicate 25th, upper boxes 75th percentile; minimum (lower error bar), maximum (upper error bar) as well as possible outliers (closed circles beyond the percentiles) are also shown. Males: noMS *N* = 10–11, MS *N* = 11; Females: noMS *N* = 9–10, MS *N* = 8–9.

In males (Fig. 5A–H) and females (Fig. 5I–P), statistical analysis revealed that absolute flexural rigidity (Fig. 5A/I), relative flexural rigidity (Fig. 5B/J), bone mineral density (Fig. 5E/M), bone volume vs. tissue volume (Fig. 5F/N), bone volume (Fig. 5G/O) and tissue volume (Fig. 5H/P) of the fractured callus were comparable between groups.

**Fig. 6:**
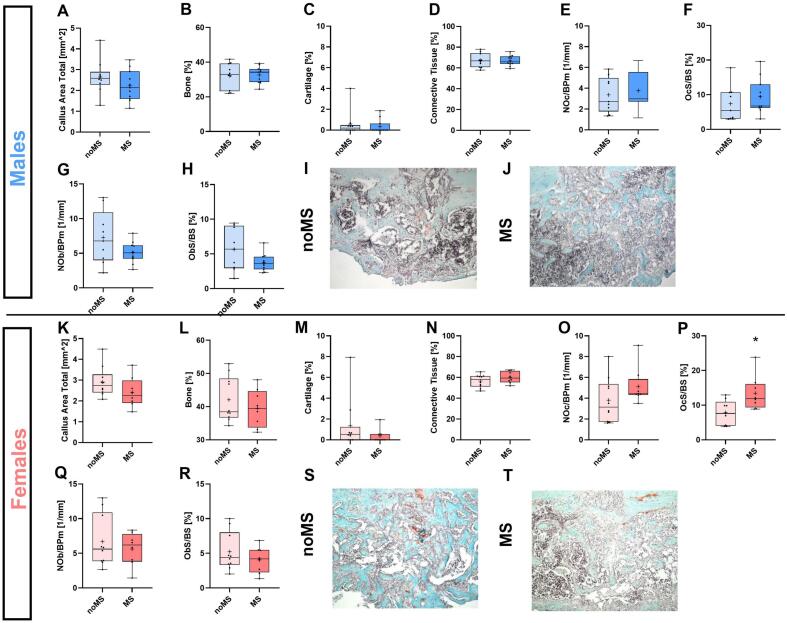
Impact of maternal separation (MS) on callus formation and bone cells during the remodeling phase of fracture healing (day 21 post-fracture). (A–H) Males. (I–P) Females. (A/I) Total callus area. (B/J) % of bone in the callus. (C/K) % of cartilage in the callus. (D/L) % of connective tissue in the callus. (E/M) Number of osteoclasts per bone perimeter (NOc/BPm). (F/N) Osteoclast surface per bone surface (OcS/BS). (G/O) Number of osteoblasts per bone perimeter (NOb/BPm). (H/P) Osteoblast surface per bone surface (ObS/BS). (I-J/S-T) Representative histological images showing cartilage (blue) and bone (red) regions in the callus stained with Safranin O. Data are presented as box-plots including individual values, with solid line representing the median and plus sign representing the mean for each data-set. Lower boxes indicate 25th, upper boxes 75th percentile; minimum (lower error bar), maximum (upper error bar) as well as possible outliers (closed circles beyond the percentiles) are also shown. * ≤ *P* 0.05 versus noMS condition. Males: noMS *N* = 9–10, MS *N* = 11; Females: noMS *N* = 9–10, MS *N* = 8.

In males, statistical analysis revealed that the total callus area (Fig. 6A), the percentage of the bone (Fig. 6B), cartilage (Fig. 6C) and connective tissue (Fig. 6D) of the callus were comparable between groups. Statistical testing of histological analysis revealed that the number of osteoclasts (Fig. 6E) and osteoblasts (Fig. 6G) per bone perimeter and osteoclast surface (Fig. 6F) and osteoblast surface (Fig. 6H) per bone surface were equivalent among the groups. In females, statistical analysis revealed that the total callus area (Fig. 6K), the percentage of the bone (Fig. 6L), cartilage (Fig. 6M) and connective tissue (Fig. 6N) of the callus were comparable between groups. Statistical testing of histological analysis revealed that the number of osteoclasts (Fig. 6O) and osteoblasts (Fig. 6Q) per bone perimeter and osteoblast surface (Fig. 6R) per bone surface were consistent between groups. Osteoclast surface per bone surface was significantly increased in MS mice (Fig. 6P; Student's *t*-test: *P* = 0.014). To ensure that effects on fracture healing were not mediated by differences in general locomotor activity, 24-h home cage activity was assessed directly after surgery. Importantly, no differences between noMS and MS mice were measured for either sex (Fig. S1C/D).

## 4 Discussion

While both, ELS and bone fractures are quite common in our society and are acknowledged as risk factors for several long-term health problems, the effects of a combination of both has not been examined yet. In this study, we used MS as a mouse model to investigate the influence of ELS on fracture healing in a sex-specific manner. Our data shows that while the early inflammatory phase (3 h post-fracture) of fracture healing is affected in males but not females by preceding MS, indicated by decreased levels of several pro- and anti-inflammatory cytokines, the later phases assessed in our study (10d and 21d post-fracture) and the overall outcome are not altered.

The fact that stress can influence inflammation is widely reported in literature. For instance, Baumeister et al. described elevated baseline levels of the pro-inflammatory cytokines IL-6 and TNF-α in individuals with a history of ELS (Baumeister et al., 2016). Another study reported that childhood maltreatment increases the risk for inflammation in adulthood, indicated by increased levels of C-reactive protein, thereby leading to associated health problems (Danese et al., 2007). In an earlier own study, we found that while chronic stress during adulthood alone increased systemic inflammatory cytokine concentrations, a combination of stress and bone fracture decreased inflammatory cytokines compared to the respective non-stressed control mice (Haffner-Luntzer et al., 2019). However, in contrast to our earlier studies on chronic adult stress and fracture, early cytokine alterations due to ELS did not translate into disturbed later phases of fracture healing. This is quite unexpected considering that cytokines like TNF-α, IFN-γ and IL10 are important regulators of bone formation, being for example involved in osteoblast differentiation and function (Ono and Takayanagi, 2017). Moreover, a down-regulation of B cell-derived IL-10 secretion has been associated with delayed fracture healing (Yang et al., 2015), and stress-related TNF-α (Miller et al., 2019) is also known to play a key role in successful fracture healing (Karnes et al., 2015).

Although further studies are clearly warranted, there are different possible explanations for the fact that the early alterations during fracture healing did not translate into disturbed callus formation, one being mechanisms in the bone tissue or the immune system that compensate for reduced cytokine levels. This hypothesis is supported by the fact that both pro- and anti-inflammatory cytokines were down-regulated. Another reason could be the existence of a threshold in cytokine levels, which is not reached to induce subsequent structural consequences. Furthermore, fracture healing is a complicated interaction of several cells and cytokines, and it is not only influenced by biological but also biomechanical factors. Male mice, which displayed stronger MS-associated cytokine alterations than female mice, also showed an MS-induced increase in general locomotion in the OF/NO Test. The elevated locomotor activity observed in MS males upon exposure to a novel arena is unlikely to reflect generalized hyperactivity. Rather, this behavioral pattern is more interpreted as an anxiolytic-like phenotype. This interpretation aligns with the match-mismatch hypothesis (Nederhof and Schmidt, 2012), which postulates that individuals exposed to repeated adverse experiences during early life develop an enhanced capacity to cope with stressful challenges later in life. Accordingly, ELS may have preconditioned MS males, specifically, to respond to novel, potentially threatening environments with reduced anxiety. This interpretation is further supported by the observation that 24-hour home cage locomotion, assessed on the day of fracture, did not significantly differ between MS and noMS male mice. Furthermore, the comparable levels of post-fracture home cage activity between groups effectively rule out differential activity-dependent mechanical loading as a confounding variable in fracture healing outcomes. Both might influence fracture healing in opposite directions, leading to null effects on later fracture healing phases.

It is generally interesting that only male MS mice seem to be affected by local changes at the fracture site, while systemic changes were inherent for both males and females. Even though further studies are needed to elude the exact mechanism, it is widely known that sex can influence the immune response after trauma (Choudhry et al., 2007; Molitoris et al., 2024). Interestingly, our group could show in a previous study that when looking at consequences on bone phenotype, female mice are more susceptible to ELS than male mice (Schimmele et al., 2025; Gebauer et al., 2025). Since the increased osteoclast surface per bone surface in our MS females at day 21 is also detectable in MS females without a bone fracture (Schimmele et al., 2025), we hypothesize that this effect is rather stress-related than being an indicator for an altered healing process.

Our study has several strengths and limitations: strengths are that we analyzed the effects of ELS on fracture healing in a sex-specific manner and that we both evaluated mouse behavior and fracture healing outcome. Furthermore, we used an external fixator model for our bone fracture, providing not only better rotational stability and standardization compared to intramedullary nail fixation, but also causing less damage to the bone marrow, which is essential for osteoimmunology (Rontgen et al., 2010; Gao et al., 2023). While we have previously used the MS model to investigate effects on bone (Schimmele et al., 2025; Gebauer et al., 2025), it is possible that another ELS model might yield different results, considering that different types of maltreatment can lead to different manifestations in adulthood (Murthy and Gould, 2018), and a combination of additional stressors like infection or malnutrition might lead to a stronger bone phenotype and, therefore, an impairment on fracture healing. This, however, remains speculation, as there are currently no other studies connecting ELS with fracture healing.

In conclusion, our results indicate that MS modulates the early inflammatory phase of fracture healing in a sex-specific manner but does not alter the overall fracture healing outcome. Further studies would be needed to elucidate underlying mechanisms. Furthermore, clinical data could be collected to analyze this in the human scenario.

## CRediT authorship contribution statement

**Dorothea Gebauer:** Writing – review & editing, Writing – original draft, Visualization, Methodology, Investigation, Formal analysis, Data curation. **Tamara Schimmele:** Writing – review & editing, Writing – original draft, Visualization, Methodology, Investigation, Formal analysis, Data curation. **Dominik Langgartner:** Writing – review & editing, Methodology. **Jana Bleher:** Writing – review & editing, Investigation. **Anita Ignatius:** Writing – review & editing, Funding acquisition, Conceptualization. **Stefan O. Reber:** Writing – review & editing, Writing – original draft, Supervision, Project administration, Funding acquisition, Data curation, Conceptualization. **Melanie Haffner-Luntzer:** Writing – review & editing, Writing – original draft, Supervision, Project administration, Funding acquisition, Data curation, Conceptualization.

## Funding

This study was supported by the Collaborative Research Centre CRC1149 (funded by the 10.13039/501100001659German Research Foundation, Project 251293561).

## Declaration of competing interest

The authors declare that they have no known competing financial interests or personal relationships that could have appeared to influence the work reported in this paper.

## Data Availability

Data will be made available on request.
